# Epidemiology of clinically significant migraine in Israel: a retrospective database study

**DOI:** 10.1186/s10194-025-01961-0

**Published:** 2025-02-03

**Authors:** A. Shifrin, E. Domany, M. Tirosh, D. Davidovici, S. Vinker, I. Forschner, A. Israel

**Affiliations:** 1 Department of Neurology, Rambam Medical Center, Haifa, Israel; 2 Pfizer Pharmaceuticals Israel, Ltd, Herzliya, Israel; 3 Leumit Research Institute, Leumit Health Services, Tel Aviv, Israel; 4 The Institute for Pain Medicine, Rambam Medical Center, Haifa, Israel; 5 Department of Epidemiology and Preventive Medicine, School of Public Health, Faculty of Medicine, Tel Aviv University, Tel Aviv, Israel

**Keywords:** Migraine, Migraine prevalence, Migraine incidence, Migraine epidemiology, Israel

## Abstract

**Background:**

Epidemiological studies on migraine are valuable for tracking disease trends, identifying risk factors, and informing treatment strategies. This study assessed the prevalence and annual incidence of clinically significant migraine in Israel from 2017 to 2022, with analyses stratified by age, sex, socioeconomic status, and district. Additionally, we compared relevant characteristics between adult migraine and non-migraine members of Leumit Healthcare Services (LHS), a national health provider in Israel.

**Methods:**

This retrospective study used LHS electronic health records to evaluate migraine prevalence and annual incidence from 2017 to 2022 among adult LHS members. Clinically significant migraine patients were identified using stringent criteria, including repeated diagnostic codes for migraine, confirmation by a neurologist, or the use of migraine-specific therapies. Each migraine patient was matched 1:1 with a control individual of similar age, sex, socioeconomic status, and ethnic background.

**Results:**

The prevalence of clinically significant migraine increased from 4.5% in 2017 to 5.2% in 2022, with significantly higher rates in women compared to men (8% vs. 2.4% in 2022). The mean age of migraine patients was 46.8 years in 2022. The annual incidence of migraine in 2022 was 43 per 10,000 individuals over 18, with approximately 75% of new cases occurring in women, with a mean age of 36.5 years. The annual incidence of migraine slightly decreased over the period. Approximately two-thirds of new patients were diagnosed by neurologists, with only 19% diagnosed by family physicians. Compared to a matched control population, migraine patients showed a higher prevalence of low body mass index (BMI) and higher diastolic blood pressure (BP). Additionally, distinct differences in laboratory findings were observed among migraine patients, notably lower glucose and hemoglobin A1c levels, lower rate of microalbuminuria, with higher hemoglobin, which may be associated with migraine pathophysiology.

**Conclusion:**

This study provides a detailed epidemiological and clinical profile of patients with clinically significant migraine in LHS from 2017 to 2022. Notable trends include higher rates of migraine among patients with lower BMI, higher diastolic BP, lower glucose, and higher hemoglobin, suggesting potential modifiable risk factors.

## Introduction

Migraine is a prevalent, often debilitating neurologic disease characterized by recurrent attacks of headache [1]. A recent estimation from the Global Burden of Disease study reported a global age-standardized prevalence of migraine in 2021 of 14.2% [2]. This study also highlighted substantial regional and national variations in migraine burden, underscoring the importance of understanding migraine epidemiology within specific healthcare systems and populations. In the United States (US) alone, approximately 39 million individuals are affected by migraine [3], with a notably higher prevalence among women compared to men [4]. The prevalence of migraine demonstrates an age-dependent pattern, peaking around age 40 before declining in older adulthood [5]. Given its extensive impact, migraine is the most disabling headache disorder, and ranks as the leading cause of disability among individuals under 50 [6]. Individuals with migraine frequently experience a diminished quality of life, reduced workplace productivity, and limited participation in social and recreational activities [5–7].

With migraine prevalence peaking among individuals aged 35–49, the disability associated with migraine significantly affects what are typically the most productive years of life [8–10]. The economic burden is also substantial, with an estimated annual cost of $36 billion in the US due to healthcare expenses and lost productivity [11]. Patients experiencing moderate pain may have some limitations in their activities, whereas severe migraine pain can be profoundly incapacitating [12]. Approximately 80% of migraine patients report impairment or an inability to function normally during attacks, with over half (53%) experiencing severe impairment and/or requiring bedrest [13]. The disability level associated with migraine has been found comparable to that of other serious conditions, such as acute myocardial infarction, dementia, and moderate multiple sclerosis [14].

Migraine management typically occurs in primary care settings, specialty neurology clinics, and emergency rooms [15]. Compared to individuals without migraine, those affected by the condition tend to utilize healthcare resources more frequently, seeking treatment from primary care providers and emergency departments (EDs) at increased rates [11]. Acute migraine treatments include non-steroidal anti-inflammatory drugs (NSAIDs), paracetamol, antiemetics, ergotamine, and triptans [16]. Prophylactic treatments focus on calcitonin gene-related peptide (CGRP) inhibitors, antidepressants, beta-blockers, and antiepileptics [17].

While the global burden of migraine has been extensively studied, understanding its prevalence, incidence, and clinical characteristics across diverse countries and healthcare systems remains scientifically valuable. Variations in healthcare system structure, population demographics, and access to care significantly influence the reported burden of migraine and its management. Despite the availability of effective treatments, many healthcare systems face challenges in the efficient diagnosis and management of migraine. Moreover, studies focusing specifically on clinically significant migraine—severe cases documented in clinical settings—remain limited.

This study provides valuable data on the epidemiology of clinically significant migraine within Israel’s healthcare system, which offers unique advantages for such research. Israel’s universal healthcare system ensures equitable access to medical care, enabling a comprehensive evaluation of migraine burden with minimal bias from healthcare accessibility—an issue commonly encountered in other settings. Leumit Healthcare Services (LHS), one of Israel’s four national health providers, serves approximately 730,000 citizens, representing about 7.5% of the country’s population. The demographic composition of LHS members broadly reflects that of Israel, with a slight overrepresentation of minority populations, including Arab and Ultra-Orthodox Jewish communities, as well as medium-to-low socioeconomic status (SES) groups. This ensures that individuals at greater risk of health disparities are appropriately represented in the population study.

LHS's centrally managed electronic health records system, rigorously maintained for over two decades, provides a robust and comprehensive dataset for epidemiological research. This robust dataset enables the identification of clinically significant migraine using stringent criteria, notably neurologist-confirmed diagnoses, treatment patterns, and comorbidities. These features allow for detailed analyses of medication use, healthcare service utilization, and the demographic and clinical profiles of patients in a real-world, population-based cohort. Prior studies have demonstrated its utility for investigating population health trends and the burden of disease in diverse subgroups [18].

By leveraging the strengths of Israel’s healthcare system and the LHS EHR database, this study fills critical gaps in the literature, offering insights into the burden and management of clinically significant migraine in a universal healthcare setting. It aims to describe the prevalence, incidence, and clinical characteristics of migraine in a large, real-world cohort, providing a foundation for future research and improved management strategies.

## Methods

### Dataset

This study involved a retrospective analysis of the database from Leumit Healthcare Services (LHS), one of the four national healthcare providers in Israel. All members have uniform health insurance coverage, ensuring equitable access to healthcare services. LHS maintains a comprehensive, centrally managed electronic health record (EHR) system that stores and continuously updates information on individual characteristics, medical diagnoses, healthcare encounters, hospital admissions, and laboratory test results. Diagnoses are coded according to the International Classification of Diseases, Ninth Revision (ICD-9) system, with previous assessments confirming the accuracy of chronic diagnoses recorded in the registry [18]. Approval for the study was obtained from the Institutional Review Board (IRB) under approval number 0008–23-LEU.

### Study design

This retrospective study analyzed the electronic health records (EHR) of Leumit Healthcare Services (LHS) members to examine the prevalence and incidence of migraine over a six-year period (2017–2022). Migraine prevalence was calculated annually, with any LHS member who met the inclusion criteria for clinically significant migraine during that year being included in the analysis.

Annual incidence was defined as the number of patients who received their first-ever diagnosis of migraine during a given year. Data from prior years, dating back to 2001, were used to confirm that individuals had no prior documented diagnosis of migraine in the LHS EHR. This ensures that incident cases reflect true first diagnoses rather than previously documented cases re-entering the healthcare system.

Migraine prevalence and annual incidence were stratified by age, sex, ethnic category, regional district, and socioeconomic status (SES). Additionally, relevant patient characteristics were compared between individuals with migraine and a matched control group of individuals with no migraine diagnosis.

### Study population

The study population included current and former Leumit Healthcare Services (LHS) members aged 18 years or older in each year of analysis. Only members with active membership during the year under consideration were included. Demographic and clinical characteristics of migraine patients were described using descriptive statistics, covering factors such as age, sex, ethnic category, regional district, and socioeconomic status (SES). A control cohort, matched on these demographic factors, was used for comparison.

### Criteria for migraine case identification

Migraine patients were identified using stringent criteria to ensure high specificity, possibly at the expense of sensitivity, to include only individuals with a strong likelihood of having migraine. The criteria were as follows:

- At least two documented instances of migraine as a chronic diagnosis in the outpatient setting.
- A hospital or emergency room (ER) ICD-9 code for migraine.
- Diagnosis confirmation by a neurology specialist.
- Use of migraine-specific therapies, such as triptans or CGRP inhibitors.

No exclusion criteria were applied in this study.

### Data collection

Data collection was automated using structured query language (SQL) and Python scripts to extract information from the electronic health records (EHR) stored in the LHS data warehouse. All data were de-identified before analysis, with identifying information encoded to ensure patient confidentiality. Migraine patients were matched 1:1 with control individuals—LHS members of similar age, sex, and socioeconomic status (SES). Patients' geocoded residential addresses were used to determine socioeconomic status (SES) levels on a scale from 1 (lowest) to 20 (highest), based on the Points Location Intelligence® database, which is highly correlated with SES measures provided by the Israeli Central Bureau of Statistics (CBS). Residential addresses were also utilized to classify LHS members into three demographic groups: the general population, Ultra-Orthodox Jews, and individuals of Arab descent, using established geodemographic methodologies validated in prior studies. Additionally, relevant individual characteristics and laboratory results were extracted from the EHR for further analysis.

Data for BMI, blood pressure (BP), and physical activity were extracted from the EHR of LHS patients. These measures are documented by treating physicians or nurses during routine medical encounters. For each patient, the most recent available measurements were used for analysis. While annual check-ups are not mandatory for all LHS members, national quality measures ensure regular documentation of BMI and BP by healthcare providers.

### Statistical analysis

Descriptive statistics, including mean, standard deviation, and frequency were used to analyze the demographic and clinical characteristics of both the patients with migraine and their control cohort matched for age and sex. Statistical analyses were conducted using R Version 4.4.1. Continuous variables were compared using the two-sample t-test, while categorical variables were assessed using Fisher's exact test.

## Results

Table 1 summarizes the prevalence of clinically significant migraine for each year from 2017 to 2022. The prevalence increased from 4.5% in 2017 to 5.2% in 2022.

**Table 1:** Prevalence of clinically significant migraine by year from 2017 to 2022

Year	Number of migraines	Number of patients	Prevalence (%)
2017	22 165	489 532	4.5
2018	22 936	487 266	4.7
2019	23 395	485 921	4.8
2020	23 690	481 329	4.9
2021	24 063	473 631	5.1
2022	24 311	468 433	5.2

**Fig. 1:**
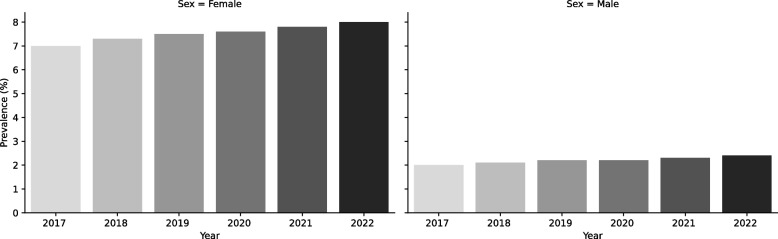
Prevalence of clinically significant migraine by year and sex

Figure 1 illustrates the prevalence of clinically significant migraine by year and sex. Across all years, migraine prevalence was consistently higher in females than in males. Both sexes showed an overall increase in prevalence during the study period.

**Fig. 2:**
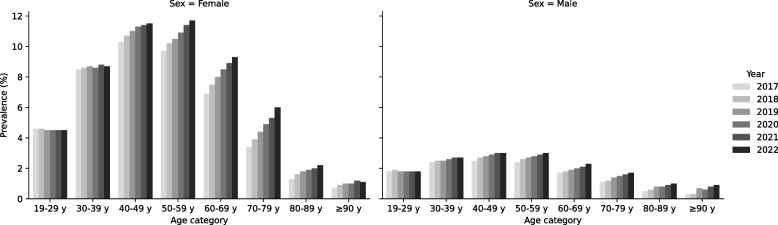
Prevalence of clinically significant migraine by year, sex, and age category

Figure 2 displays the prevalence of clinically significant migraine stratified by year, sex, and age category. The highest prevalence was observed among females aged 50–59 years (11.7% in 2022), followed by females aged 40–49 years (11.5% in 2022).

**Fig. 3:**
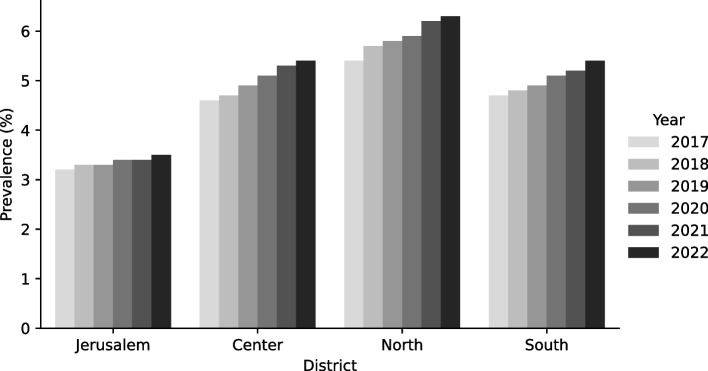
Prevalence of clinically significant migraine by year and regional district

Figure 3 presents the prevalence of clinically significant migraine by year and regional district. The Northern district of Israel consistently exhibited the highest prevalence across all years from 2017 to 2022.

Table 2 presents data from 2017 to 2022 on various parameters related to migraine patients. The prevalence of migraine increased from 2017 to 2022 (from 22 165 to 24 311), with the majority of diagnosed patients being female. The mean age of diagnosed patients also gradually increased over this period. The highest prevalence of migraine was observed among individuals in their fourth decade of life and in the Northern region. The highest prevalence of migraine was seen in the 9–11 SES category. Most patients belonged to the General ethnic category, with the lowest prevalence in the Jewish Ultra-Orthodox category. The highest prevalence was found in the normal BMI category compared to overweight, obese, or underweight categories.

**Table 2:** Characteristics of patients with clinically significant migraine by year

Characteristic		2017	2018	2019	2020	2021	2022
Number of patients		22 165	22 936	23 395	23 690	24 063	24 311
Sex	Female (%)	17 222 (77.7)	17 745 (77.4)	18 123 (77.5)	18 334 (77.4)	18 552 (77.1)	18 795 (77.3)
	Male (%)	4941 (22.3)	5189 (22.6)	5270 (22.5)	5354 (22.6)	5509 (22.9)	5516 (22.7)
Age (mean ± SD)		44.0 ± 14.4	44.5 ± 14.6	45.1 ± 14.8	45.7 ± 14.9	46.2 ± 15.1	46.8 ± 15.2
Age category, y (%)	19–29	4171 (18.8)	4164 (18.2)	4043 (17.3)	3948 (16.7)	3816 (15.9)	3716 (15.3)
	30–39	4897 (22.1)	4949 (21.6)	4968 (21.2)	4897 (20.7)	4950 (20.6)	4866 (20.0)
	40–49	5194 (23.4)	5345 (23.3)	5416 (23.2)	5406 (22.8)	5363 (22.3)	5334 (21.9)
	50–59	4416 (19.9)	4581 (20.0)	4677 (20.0)	4775 (20.2)	4924 (20.5)	4982 (20.5)
	60–69	2592 (11.7)	2801 (12.2)	3026 (12.9)	3229 (13.6)	3375 (14.0)	3531 (14.5)
	70–79	723 (3.3)	869 (3.8)	1004 (4.3)	1162 (4.9)	1334 (5.5)	1558 (6.4)
	80–89	150 (0.7)	199 (0.9)	226 (1.0)	239 (1.0)	260 (1.1)	282 (1.2)
	≥ 90	22 (0.1)	28 (0.1)	35 (0.2)	34 (0.1)	41 (0.2)	42 (0.2)
SES (%)	1–20 (mean ± SD)	9.1 ± 3.6	9.0 ± 3.6	9.0 ± 3.6	9.0 ± 3.6	8.9 ± 3.6	8.9 ± 3.6
	Data not available	1306 (5.9)	1438 (6.3)	1487 (6.4)	1508 (6.4)	1641 (6.8)	1633 (6.7)
SES category (%)	0–5	3858 (18.5)	4086 (19.0)	4247 (19.4)	4365 (19.7)	4535 (20.2)	4666 (20.6)
	6–8	5064 (24.3)	5156 (24.0)	5274 (24.1)	5389 (24.3)	5453 (24.3)	5549 (24.5)
	9–11	6626 (31.8)	6860 (31.9)	6953 (31.7)	6992 (31.5)	7001 (31.2)	7033 (31.0)
	≥ 12	5311 (25.5)	5396 (25.1)	5434 (24.8)	5436 (24.5)	5433 (24.2)	5430 (23.9)
	Data not available	1306 (5.9)	1438 (6.3)	1487 (6.4)	1508 (6.4)	1641 (6.8)	1633 (6.7)
Region (%)	Center	6077 (27.4)	6233 (27.2)	6369 (27.2)	6445 (27.2)	6522 (27.1)	6584 (27.1)
	Jerusalem	3153 (14.2)	3238 (14.1)	3365 (14.4)	3451 (14.6)	3547 (14.7)	3631 (14.9)
	North	6585 (29.7)	6928 (30.2)	7045 (30.1)	7124 (30.1)	7262 (30.2)	7309 (30.1)
	South	6350 (28.6)	6537 (28.5)	6616 (28.3)	6670 (28.2)	6732 (28.0)	6787 (27.9)
Ethnic category (%)	General	15 236 (68.7)	15 562 (67.9)	15 713 (67.2)	15 764 (66.5)	15 803 (65.7)	15 853 (65.2)
	Arab	4770 (21.5)	5074 (22.1)	5260 (22.5)	5452 (23.0)	5712 (23.7)	5822 (24.0)
	Jewish Ultra-orthodox	2159 (9.7)	2300 (10.0)	2422 (10.4)	2474 (10.4)	2548 (10.6)	2636 (10.8)
Duration of migraine diagnosis (%)	0–2	3280 (14.8)	3396 (14.8)	3059 (13.1)	2647 (11.2)	2769 (11.5)	2758 (11.3)
	≥ 3	18 885 (85.2)	19 540 (85.2)	20 336 (86.9)	21 043 (88.8)	21 294 (88.5)	21 553 (88.7)
BMI (kg/m^2^) (%)	(mean ± SD)	27.0 ± 5.6	27.1 ± 5.6	27.2 ± 5.7	27.4 ± 5.7	27.4 ± 5.8	27.4 ± 5.8
	Data not available	2571 (11.6)	2341 (10.2)	2078 (8.9)	2072 (8.8)	2186 (9.1)	1989 (8.2)
BMI category (%)	< 18.5 Underweight	528 (2.7)	545 (2.7)	544 (2.6)	515 (2.4)	555 (2.5)	609 (2.7)
	18.5–24.9 Normal	7226 (36.9)	7419 (36.0)	7584 (35.6)	7547 (34.9)	7589 (34.7)	7742 (34.7)
	25–29.9 Overweight	6711 (34.3)	7101 (34.5)	7318 (34.3)	7447 (34.5)	7475 (34.2)	7484 (33.5)
	≥ 30 Obese	5129 (26.2)	5530 (26.9)	5871 (27.5)	6109 (28.3)	6258 (28.6)	6487 (29.1)
	Data not available	2571 (11.6)	2341 (10.2)	2078 (8.9)	2072 (8.8)	2186 (9.1)	1989 (8.2)
Migraine diagnosis by family physician during the year (%)		5548 (25.0)	5486 (23.9)	5291 (22.6)	4854 (20.5)	4803 (20.0)	4660 (19.2)
Migraine diagnosis by pain physician (%)		56 (0.3)	60 (0.3)	58 (0.3)	58 (0.2)	62 (0.3)	63 (0.3)
Criterion outpatient diagnosis by neurologist (%)		14,769 (66.6)	15 136 (66.0)	15 436 (66.0)	15 631 (66.0)	15 916 (66.1)	16 227 (66.7)
Criterion at least 2 chronic migraine diagnoses (%)		17 741 (80.0)	18 281 (79.7)	18 752 (80.2)	19 027 (80.3)	19 240 (80.0)	19 492 (80.2)
Criterion hospital diagnosis (%)		-	172 (0.8)	309 (1.3)	416 (1.8)	492 (2.0)	589 (2.4)
Criterion inpatient diagnosis (%)		-	163 (0.7)	292 (1.3)	366 (1.5)	439 (1.8)	537 (2.2)
Criterion migraine triptan use (%)		4246 (19.2)	4237 (18.5)	4151 (17.7)	3939 (16.6)	4156 (17.3)	3990 (16.4)
Criterion migraine CGRP use (%)		-	-	-	24 (0.1)	132 (0.6)	232 (1.0)

*CGRP* calcitonin gene-related peptide, *SD* standard deviation, *SES* socioeconomic status, *y* year

Migraine diagnoses were most commonly made by neurologists in an outpatient setting, followed by family physicians, and, less frequently, by pain specialists. The most prevalent criterion for including a migraine case in the cohort was having at least two chronic migraine diagnoses, followed by diagnosis by a neurologist and triptan use. Few patients were included based on hospital or inpatient diagnoses or CGRP inhibitor use. The prevalence of CGRP inhibitor use for migraine treatment showed an increase from 0.1% in 2020 to 1.0% in 2022.

The annual incidence of migraine was highest in 2018 and subsequently declined through 2022 (Table 3). Females exhibited a higher incidence and prevalence of clinically significant migraine throughout the study years, with some variations between 70.3% of cases in 2021 and 75.3% in 2017. Conversely, males represented 24.7% of cases in 2017, increasing slightly to 29.7% in 2021. The mean (± SD) age of new migraine patients remained relatively stable throughout the study period. The highest incidence of migraine was observed in the 19–29 age group, followed by the 30–39 age group. The lowest incidence was seen in the 80–89 and 90 + age groups, each with less than 1% of cases. The majority of annual incidence cases were categorized as having a duration of disease of 0–2 years. The mean (± SD) socioeconomic status (SES) remained relatively consistent over the years. The highest incidence of migraine occurred in the North region followed by the South, Center, and Jerusalem regions.

**Table 3:** Annual incidence and characteristics of new patients with clinically significant migraine

Year		2017	2018	2019	2020	2021	2022
**Number**		2035	2108	1735	1629	1765	1685
Sex	Female (%)	1533 (75.3)	1534 (72.8)	1299 (74.9)	1173 (72.0)	1241 (70.3)	1250 (74.2)
	Male (%)	502 (24.7)	574 (27.2)	436 (25.1)	456 (28.0)	524 (29.7)	435 (25.8)
Age (mean ± SD)		36.4 ± 14.2	36.8 ± 14.7	36.8 ± 14.9	36.0 ± 14.4	37.0 ± 14.8	36.5 ± 14.8
Age category, y (%)	19–29	798 (39.2)	817 (38.8)	687 (39.6)	663 (40.7)	671 (38.0)	690 (41.0)
	30–39	460 (22.6)	474 (22.5)	391 (22.5)	363 (22.3)	394 (22.3)	364 (21.6)
	40–49	405 (19.9)	385 (18.3)	308 (17.8)	293 (18.0)	352 (19.9)	295 (17.5)
	50–59	223 (11.0)	259 (12.3)	197 (11.4)	192 (11.8)	203 (11.5)	192 (11.4)
	60–69	99 (4.9)	116 (5.5)	108 (6.2)	84 (5.2)	88 (5.0)	93 (5.5)
	70–79	42 (2.1)	44 (2.1)	31 (1.8)	27 (1.7)	45 (2.6)	38 (2.3)
	80–89	7 (0.3)	13 (0.6)	10 (0.6)	6 (0.4)	11 (0.6)	12 (0.7)
	≥ 90	1 (< 0.1)	0	3 (0.2)	1 (0.1)	1 (0.1)	1 (0.1)
SES (1–20) (%)	(mean ± SD)	8.6 ± 3.6	8.6 ± 3.6	8.6 ± 3.6	8.3 ± 3.6	8.3 ± 3.6	8.3 ± 3.5
	Data not available	163 (8.0)	212 (10.1)	167 (9.6)	136 (8.4)	224 (12.7)	149 (8.8)
Region (%)	Center	544 (26.7)	532 (25.2)	465 (26.8)	407 (25.0)	444 (25.2)	453 (26.9)
	Jerusalem	302 (14.8)	338 (16.0)	312 (18.0)	280 (17.2)	285 (16.1)	269 (16.0)
	North	628 (30.9)	669 (31.7)	507 (29.2)	478 (29.3)	543 (30.8)	485 (28.8)
	South	561 (27.6)	569 (27.0)	451 (26.0)	464 (28.5)	493 (27.9)	478 (28.4)
Ethnic category (%)	Arab	596 (29.3)	639 (30.3)	497 (28.7)	508 (31.2)	564 (32.0)	504 (29.9)
	General	1239 (60.9)	1228 (58.3)	1010 (58.2)	929 (57.0)	992 (56.2)	968 (57.5)
	Jewish Ultra-orthodox	200 (9.8)	241 (11.4)	228 (13.1)	192 (11.8)	209 (11.8)	213 (12.6)
Duration of migraine diagnosis (%)	0–2	1851 (91.0)	1937 (91.9)	1588 (91.5)	1448 (88.9)	1611 (91.3)	1557 (92.4)
	≥ 3	184 (9.0)	171 (8.1)	147 (8.5)	181 (11.1)	154 (8.7)	128 (7.6)

*NA* not applicable, *SD* standard deviation, *SES* socioeconomic status, *y* year

Table 4 displays a comparison of the demographic characteristics of patients with clinically significant migraine and the matched control cohort. In accordance with the matching criteria used for the control cohort, no significant differences were observed in mean age (± SD), age category, ethnic category, SES status, or SES category between migraine and control participants. However, some regional differences were identified: a significantly higher proportion of patients in the control cohort were from the Jerusalem and South regions compared to the migraine cohort (*p* < 0.001 and *p* = 0.038, respectively). Conversely, significantly more participants in the migraine cohort were from the North region compared to the control cohort (*p* < 0.001).

**Table 4:** Comparison of the demographic characteristics between patients with clinically significant migraine and the matched control cohort

Characteristic		Case	Control	*p*-value	Odds ratio
**Number of patients**		23 143	23 143	NA	NA
Age (mean ± SD)		47.1 ± 15.1	47.0 ± 15.5	0.576	
Age category, y (%)	19–29	3364 (14.5)	3364 (14.5)	1	1.00 [0.95 to 1.05]
	30–39	4568 (19.7)	4568 (19.7)	1	1.00 [0.95 to 1.05]
	40–49	5123 (22.1)	5123 (22.1)	1	1.00 [0.96 to 1.05]
	50–59	4847 (20.9)	4847 (20.9)	1	1.00 [0.96 to 1.05]
	60–69	3450 (14.9)	3450 (14.9)	1	1.00 [0.95 to 1.05]
	70–79	1499 (6.5)	1499 (6.5)	1	1.00 [0.93 to 1.08]
	80–89	254 (1.1)	254 (1.1)	1	1.00 [0.84 to 1.20]
	≥ 90	38 (0.2)	38 (0.2)	1	1.00 [0.62 to 1.61]
Region (%)	Center	6283 (27.1)	6332 (27.4)	0.616	0.99 [0.95 to 1.03]
	Jerusalem	3468 (15.0)	3825 (16.5)	< 0.001	0.89 [0.85 to 0.94]
	North	6948 (30.0)	6340 (27.4)	< 0.001	1.14 [1.09 to 1.18]
	South	6444 (27.8)	6646 (28.7%)	0.038	0.96 [0.92 to 1.00]
Ethnic category (%)	Arab	5518 (23.8)	5518 (23.8)	1	1.00 [0.96 to 1.04]
	General	15 097 (65.2)	15 097 (65.2)	1	1.00 [0.96 to 1.04]
	Jewish Ultra-orthodox	2528 (10.9)	2528 (10.9)	1	1.00 [0.94 to 1.06]
SES (1–20) (%)	(mean ± SD)	8.9 ± 3.6	8.9 ± 3.7	0.418	NA
	Data not available	1560 (6.7)	1560 (6.7)	NA	1.00 [0.93 to 1.08]
SES category (%)	0–3	967 (4.5)	967 (4.5)	1	1.00 [0.91 to 1.10]
	4–6	5492 (25.5)	5492 (25.5)	1	1.00 [0.96 to 1.04]
	7–9	4977 (23.1)	4977 (23.1)	1	1.00 [0.96 to 1.05]
	≥ 10	10 147 (47.0)	10 147 (47.0)	1	1.00 [0.96 to 1.04]
	Data not available	1560 (7.2)	1560 (7.2)	1	1.00 [0.93 to 1.08]

*NA* not applicable, *SD* standard deviation, *SES* socioeconomic status, *y* year

Table 5 presents a comparison of clinical characteristics and body measures between patients with clinically significant migraine and the matched control cohort. The migraine cohort had a statistically significant lower BMI (27.1 vs. 27.4, *p* < 0.001), fewer obese patients (OR 0.94; *p* < 0.001), higher diastolic BP (75.1 vs. 74.6, *p* < 0.001), and a higher incidence of stage 1 hypertension (OR 1.08; *p* = 0.015) compared to the control cohort. No significant difference in physical activity was observed between the migraine and control cohorts.

**Table 5:** Comparison of the clinical characteristics and body measures of patients with clinically significant migraine to those of the matched control cohort

Characteristic		Case	Control	*p*-value	Odds ratio	SMD
**Number of patients**		23 143	23 143	NA	NA	NA
Weight (kg) (%)	(mean ± SD)	73.6 ± 16.5	73.9 ± 17.3	0.052	NA	−0.018
	Data not available	39 (0.2)	459 (2.0)	NA	0.08 [0.06 to 0.12]	NA
Height (cm) (%)	(mean ± SD)	165 ± 9	164 ± 9	< 0.001	NA	0.051
	Data not available	85 (0.4)	618 (2.7)	NA	0.13 [0.11 to 0.17]	NA
BMI (kg/m^2^) (%)	(mean ± SD)	27.1 ± 5.6	27.4 ± 5.9	< 0.001	NA	−0.048
	Data not available	107 (0.5)	654 (2.8)	NA	0.16 [0.13 to 0.20]	NA
BMI category	< 18.5 Underweight	683 (3.0)	676 (3.01)	0.804	1.01 [0.91 to 1.13]	NA
	18.5–24.9 Normal	8247 (35.8)	7838 (34.9)	0.035	1.08 [1.04 to 1.12]	NA
	25–29.9 Overweight	7858 (34.1)	7421 (33.0)	0.012	1.09 [1.05 to 1.13]	NA
	≥ 30 Obese	6248 (27.1)	6554 (29.1)	< 0.001	0.94 [0.90 to 0.98]	NA
	Data not available	107 (0.5)	654 (2.8)	< 0.001	0.16 [0.13 to 0.20]	NA
BP systolic (mmHg) (%)	(mean ± SD)	122 ± 16	122 ± 17	0.029	NA	−0.020
	Data not available	49 (0.2)	494 (2.1)	NA	0.10 [0.07 to 0.13]	NA
BP diastolic (mmHg) (%)	(mean ± SD)	75.1 ± 9.9	74.6 ± 10.0	< 0.001	NA	0.056
	Data not available	53 (0.2)	496 (2.1)	NA	0.10 [0.08 to 0.14]	NA
BP category (%)	Hypertension 1	5145 (22.3)	4833 (21.3)	0.015	1.08 [1.04 to 1.13]	NA
	Hypertension 2	2914 (12.6)	3003 (13.3)	0.042	0.97 [0.91 to 1.02]	NA
	Normal BP	15 031 (65.1)	14 811 (65.4)	0.498	1.04 [1.00 to 1.08]	NA
	Data not available	53 (0.2)	496 (2.1)	< 0.001	0.10 [0.08 to 0.14]	NA
Physical activity (%)	1–3 h weekly	3515 (22.0)	3605 (22.0)	0.947	0.97 [0.92 to 1.02]	NA
	> 3 h weekly	1355 (8.5)	1383 (8.4)	0.936	0.98 [0.91 to 1.06]	NA
	None	5648 (35.3)	5756 (35.1)	0.762	0.98 [0.93 to 1.02]	NA
	Occasionally	5481 (34.3)	5631 (34.4)	0.833	0.97 [0.92 to 1.01]	NA
	Yes	4 (< 0.1)	11 (0.1)	0.119	0.36 [0.08 to 1.23]	NA
	Data not available	7140 (30.9)	6757 (29.2)	< 0.001	1.08 [1.04 to 1.13]	NA

*BMI* body mass index, *BP* blood pressure, *NA* not applicable, *SD* standard deviation, *SMD* standardized mean difference

Table 6 presents a comparison of common laboratory tests between patients with clinically significant migraine and the matched control cohort. The migraine cohort had statistically significantly lower glucose levels (94.4 ± 20.6 vs. 97.0 ± 25.5; *p* < 0.001),a higher rate of patients with a normal range of hemoglobin A1c (OR = 1.73; *p* < 0.001), a lower rate of microalbuminuria (OR = 0.89; *p* = 0.003), slightly lower mean estimated glomerular filtration rate (eGFR MDRD) scores (*p* < 0.001), and slightly higher hemoglobin (13.4 ± 1.5 vs. 13.3 ± 1.5 *p* < 0.001) compared to the control cohort.

**Table 6:** Comparison of common laboratory test results between patients with clinically significant migraine and the matched control cohort

Characteristic		Case	Control	*p*-value	Odds ratio	SMD
Glucose (mg/dL)	(mean ± SD)	94.4 ± 20.6	97.0 ± 25.5	< 0.001	NA	−0.112
	Data not available	126 (0.5)	887 (3.8)	NA	0.14 [0.11 to 0.17]	NA
Glucose category (%)	0–100	18 053 (78.4)	16 399 (73.7)	< 0.001	1.46 [1.40 to 1.52]	NA
	100–125	3938 (17.1)	4373 (19.7)	< 0.001	0.88 [0.84 to 0.92]	NA
	126–199	885 (3.8)	1249 (5.6)	< 0.001	0.70 [0.64 to 0.76]	NA
	≥ 200	141 (0.6)	235 (1.1)	< 0.001	0.60 [0.48 to 0.74]	NA
	Data not available	126 (0.5)	887 (3.8)	< 0.001	0.14 [0.11 to 0.17]	NA
HGB A1c (%)	(mean ± SD)	5.5 ± 0.7	5.6 ± 0.9	< 0.001	NA	−0.133
	Data not available	3321 (14.3)	5072 (21.9)	NA	0.60 [0.57 to 0.63]	NA
A1C range (%)	0–6.5	18 879 (95.2)	16 633 (92.0)	< 0.001	1.73 [1.66 to 1.81]	NA
	6.5–8	674 (3.4)	976 (5.4)	< 0.001	0.68 [0.62 to 0.75]	NA
	8–10	190 (1.0)	345 (1.9)	< 0.001	0.55 [0.46 to 0.66]	NA
	≥ 10	79 (0.4)	117 (0.7)	< 0.001	0.67 [0.50 to 0.91]	NA
	Data not available	3321 (14.4)	5072 (21.9)	< 0.001	0.60 [0.57 to 0.63]	NA
Albumin/Creatinine ratio (%)	(mean ± SD)	26.4 ± 280.3	34.2 ± 278.8	0.019	NA	−0.028
	Data not available	7897 (34.1)	9756 (42.2)	NA	0.71 [0.68 to 0.74]	NA
Macroalbuminuria (%)		170 (0.7)	199 (0.9)	0.143	0.85 [0.69 to 1.05]	NA
Microalbuminuria (%)		1293 (5.6)	1445 (6.2)	0.003	0.89 [0.82 to 0.96]	NA
**eGFR MDRD (mL/min/1.73 m**^**2**^**)**	(mean ± SD)	101 ± 26	103 ± 32	< 0.001	NA	−0.079
	Data not available	109 (0.5)	935 (4.0)	NA	0.11 [0.09 to 0.14]	NA
eGFR category (%)	G1 (normal)	15 041 (65.3)	14 935 (67.3)	0.307	1.02 [0.98 to 1.06]	NA
	G2 60–89	7383 (32.1)	6626 (29.8)	< 0.001	1.17 [1.12 to 1.22]	NA
	G3a 45–59	469 (2.0)	474 (2.1)	0.895	0.99 [0.87 to 1.13]	NA
	G3b 30–44	111 (0.5)	112 (0.5)	1	0.99 [0.75 to 1.30]	NA
	G4 15–29	17 (0.1)	41 (0.2)	0.002	0.41 [0.22 to 0.75]	NA
	G5 < 15	13 (0.1)	20 (0.1)	0.296	0.65 [0.30 to 1.37]	NA
	Data not available	109 (0.5)	935 (4.2)	< 0.01	0.11 [0.09 to 0.14]	NA
HDL cholesterol (mg/dL) (%)	(mean ± SD)	53.1 ± 12.9	52.9 ± 13	0.017	NA	0.023
	Data not available	310 (1.3)	1349 (5.8)	NA	0.22 [0.19 to 0.25]	NA
LDL cholesterol (mg/dL) (%)	(mean ± SD)	119 ± 34	118 ± 35	0.006	NA	0.026
	Data not available	318 (1.4)	1 373 (6.0)	NA	0.22 [0.19 to 0.25]	NA
HGB (g/dL) (%)	(mean ± SD)	13.4 ± 1.5	13.3 ± 1.5	< 0.001	NA	0.061
	Data not available	66 (0.3)	648 (2.8)	NA	0.10 [0.08 to 0.13]	NA

*eGFR* estimated glomerular filtration rate, *HDL* high-density lipoprotein, *HGB* hemoglobin, *LDL* low-density lipoprotein, *MDRD* modification of diet in renal disease, *NA* not applicable, *SD* standard deviation, *SMD* standardized mean difference

## Discussion

This retrospective epidemiologic study analyzed data from almost 25 000 individuals aged over 18 who were diagnosed with migraine up to 2022. During this period, we observed a steady increase in migraine prevalence, from 4.5% in 2017 to 5.2% in 2022, underscoring a growing disease burden. Migraine was more prevalent among women (8.0% vs. 2.4% in men), and the mean age of migraine patients was 46.8 years, with the highest prevalence observed in individuals in their third and fourth decades of life. Using our highly specific criteria for migraine, the annual incidence in 2022 was 3.6 cases per 1000 individuals, with approximately 75% of new cases occurring in women, whose mean age was 36.5 years.

Interestingly, despite the introduction of new migraine-specific treatments and increased awareness efforts by medical societies and pharmaceutical companies, the number of new migraine cases declined from 2035 in 2017 to 1686 in 2022. This decrease may be attributed to several factors. First, the COVID-19 pandemic likely discouraged some individuals from seeking healthcare services, impacting diagnosis rates [19]. Second, the relatively low percentage of LHS members with complementary insurance (70.9% vs. 79% in the general population in 2020) may have limited access to new-generation migraine treatments, as complementary insurance is the primary means for obtaining these therapies. Patients without access to these newer treatments might be less likely to seek or receive a formal migraine diagnosis. Additionally, most migraine awareness activities have been concentrated in central Israel, where a smaller proportion of citizens are LHS-insured, possibly impacting awareness and diagnosis rates in other regions.

The observed increase in migraine prevalence from 2017 to 2022 aligns with global trends reported from 1990 to 2019 [20]. This upward trend may be attributed to improvements in diagnostic procedures and an increased tendency for patients to seek medical care for migraine symptoms. Despite this increase, the prevalence rate in our study remains relatively low compared to other recent local and global data. Globally, the prevalence of migraine is estimated to be around 14.0% [21], and a recent study from southern Israel reported a prevalence of 7.65% (11.43% in women and 3.75% in men)[22]. This discrepancy may be explained by the lower percentage of middle-aged individuals insured with LHS, as this age group constitutes a substantial proportion of migraine patients, as well as the stringent criteria we used to identify migraine [23]. Indeed, nearly two-thirds of migraine patients identified using the stringent criteria of this study were diagnosed by neurologists, while only 19% were diagnosed by family physicians. This contrasts with previous studies, where migraine diagnoses were more commonly made by general practitioners [24].

The higher prevalence of migraine in females aligns with findings from previous studies conducted in the US, Spain, Japan, and Italy [15, 25, 26]. The mean age of patients in our migraine cohort was 47.6 years, slightly higher than the previously reported mean of 40.3 years. This difference may be attributed to the fact that migraine diagnoses sometimes remain in electronic health records even after symptoms have subsided [15]. The mean age of migraine diagnosis in our cohort, around 36.5 years, is consistent with the previously reported age of 38.2 years [27]. The annual incidence of migraine reported in a United Kingdom database study was 3.69 cases per 1000 [28], similar to the 3.6 cases per 1000 observed in our study for 2022.

Anti CGRP MAB's (Monoclonal Antibodies) were approved by the US Food and Drug Administration (FDA) in 2018 for the preventive treatment of migraine and their use in Israel began in 2019–2020 [29]. The prevalence of CGRP inhibitor use increased from 0.1% in 2020 to 1% in 2022. The higher cost of CGRP inhibitors compared to triptans may have limited their use [30]. However, CGRP inhibitor usage has gradually increased, which could be attributed to their favorable safety and tolerability profile [31] especially the aging population. A previous study suggested that CGRP inhibitors are associated with fewer side effects in older patients and in those with comorbidities and concurrent medication use [32].

To identify risk factors, we compared physical measurements and laboratory test results between individuals with migraine and matched controls. We found that patients with migraine had a lower BMI, a decreased rate of obesity, higher diastolic blood pressure (BP), higher hemoglobin levels, lower glucose levels, a higher rate of normal HbA1c (below 6.5), and lower rates of microalbuminuria compared to the control cohort. These findings align with a recent meta-analysis by Ha et al. (2024), which reported a reverse association between diabetes and migraine, further supporting the observed differences in HbA1c and glucose levels in our study [33].

Research suggests that both underweight (BMI ≤ 18) [34] and overweight (BMI ≥ 30) [35–37] are associated with an increased risk of migraine. In this study, individuals with clinically significant migraine had a higher prevalence of normal weight (BMI 18.5–24.9) and overweight (BMI 25–29.9), while obesity (BMI > 30) was more common in the control cohort. These findings underscore the complex relationship between BMI and migraine risk, highlighting the importance of addressing body weight as a modifiable factor in the diagnosis and management of migraine [38].

Previous studies have highlighted associations between migraine and hypoglycemia (low blood glucose) [39], and hypertension [40], findings that were confirmed by our study. Additionally, while past research has linked migraine with low hemoglobin (particularly in patients with iron-deficiency anemia) [41], our study found a higher average hemoglobin level among individuals with migraine.

The laboratory differences observed in this study suggest potential risk factors that may influence migraine risk in the population and could provide valuable directions for future research.

This study has several strengths, including the use of high-quality data from the electronic health records (EHR) of a national health provider in Israel, encompassing a comprehensive review of patient medical records and pharmacy purchases. The study benefits from long-term follow-up, well-ascertained outcomes, and the use of relatively recent data, enabling a robust analysis of clinically significant migraine epidemiology. Additionally, the inclusion of diverse populations, such as Arab and Ultra-Orthodox Jewish communities and individuals from medium-to-low socioeconomic status (SES) groups, ensures that subpopulations at higher risk of health disparities are adequately represented.

However, several limitations should be considered when interpreting these results. First, as a retrospective and observational study, it shares the inherent limitations of such a design, including potential confounding and bias. Second, the study lacks data on the severity and frequency of migraine symptoms, as well as information on private medication purchases made outside LHS pharmacies, which may have influenced treatment patterns. Third, crude estimates were used instead of standardized estimates, meaning that demographic differences could be confounded by the LHS population characteristics. Consequently, caution is advised when interpreting ethnic, socioeconomic, and geographical disparities.

Furthermore, while our descriptive approach provides valuable insights into migraine epidemiology, this study did not utilize regression models to investigate the associations between socio-demographics, clinical variables, and migraine prevalence. Although we mitigated potential confounding effects through careful matching of cases and controls on key variables, the absence of regression analysis limits our ability to explore these relationships in greater depth. Future studies are planned to build on these findings by using multivariable regression models to gain a deeper understanding of these associations and their potential influence on incidence trends.

Lastly, the cohort reflects migraine patients within LHS, one of four health maintenance organizations in Israel. LHS has a stronger presence in the country’s periphery and a smaller representation in central Israel, where most migraine awareness activities have been concentrated in recent years, compared to other health funds. This may limit the generalizability of the observed prevalence and incidence trends to other healthcare settings or regions.

## Conclusion

This study presents findings from a large real-world retrospective analysis conducted within a national healthcare provider from 2017 to 2022, using electronic health records and applying stringent, highly specific criteria for migraine identification, with most migraine patients diagnosed by neurologists. Over this period, incidence of clinically significant migraine showed a slight annual decrease, while prevalence increased. Migraine rates were notably higher in women than in men. Significant differences in physical measures were observed between migraine patients and matched controls, including lower BMI, lower blood glucose levels, lower hemoglobin A1c and higher incidence of hypertension 1. These findings may be related to migraine pathophysiology and suggest directions for further investigation.

## Data Availability

The data analyzed in this study is subject to the following licenses/restrictions: Access to raw patient data is restricted to researchers approved by the institutional ethics committee. Requests to access these datasets should be directed to aisrael@leumit.co.il.
